# Molecular mechanisms behind progressing chronic inflammatory dilated cardiomyopathy

**DOI:** 10.1186/s12872-015-0017-1

**Published:** 2015-03-26

**Authors:** Daiva Bironaite, Dainius Daunoravicius, Julius Bogomolovas, Sigitas Cibiras, Dalius Vitkus, Edvardas Zurauskas, Ieva Zasytyte, Kestutis Rucinskas, Siegfried Labeit, Algirdas Venalis, Virginija Grabauskiene

**Affiliations:** Dept. of Stem Cell Biology, State Research Institute, Center for Innovative Medicine, Zygimantu 9, LT01102 Vilnius, Lithuania; Department of Pathology, Forensic Medicine and Pharmacology, Vilnius University, Faculty of Medicine, Vilnius, Lithuania; Department of Integrative Pathophysiology, Universitätsmedizin Mannheim, Mannheim, Germany; Department of Physiology, Biochemistry, Microbiology and Laboratory Medicine, Vilnius University, Faculty of Medicine, Vilnius, Lithuania; Vilnius University, Faculty of Medicine, Clinic of Cardiovascular Diseases, Vilnius, Lithuania

**Keywords:** Apoptosis, Dilated cardiomyopathy, Heart, Inflammation, Necrosis

## Abstract

**Background:**

Inflammatory dilated cardiomyopathy (iDCM) is a common debilitating disease with poor prognosis that often leads to heart failure and may require heart transplantation. The aim of this study was to evaluate sera and biopsy samples from chronic iDCM patients, and to investigate molecular mechanism associated with left ventricular remodeling and disease progression in order to improve therapeutic intervention.

**Methods:**

Patients were divided into inflammatory and non-inflammatory DCM groups according to the immunohistochemical expression of inflammatory infiltrates markers: T-lymphocytes (CD3), active-memory T lymphocyte (CD45Ro) and macrophages (CD68). The inflammation, apoptosis, necrosis and fibrosis were investigated by ELISA, chemiluminescent, immunohistochemical and histological assays.

**Results:**

The pro-inflammatory cytokine IL-6 was significantly elevated in iDCM sera (3.3 vs. 10.98 μg/ml; *P* < 0.05). Sera levels of caspase-9, −8 and −3 had increased 6.24-, 3.1- and 3.62-fold, (*P* < 0.05) and only slightly (1.3-, 1.22- and 1.03-fold) in biopsies. Significant release of Hsp60 in sera (0.0419 vs. 0.36 ng/mg protein; *P* < 0.05) suggested a mechanistic involvement of mitochondria in cardiomyocyte apoptosis. The significant MMP9/TIMP1 upregulation in biopsies (0.1931 - 0.476, *P* < 0.05) and correlation with apoptosis markers show its involvement in initiation of cell death and ECM degradation. A slight activation of the extrinsic apoptotic pathway and the release of hsTnT might support the progression of chronic iDCM.

**Conclusions:**

Data of this study show that significant increase of IL-6, MMP9/TIMP1 and caspases-9, −8, −3 in sera corresponds to molecular mechanisms dominating in chronic iDCM myocardium. The initial apoptotic pathway was more activated by the intramyocardial inflammation and might be associated with extrinsic apoptotic pathway through the pro-apoptotic Bax. The activated intrinsic form of myocardial apoptosis, absence of necrosis and decreased fibrosis are most typical characteristics of chronic iDCM. Clinical use of anti-inflammatory drugs together with specific anti-apoptotic treatment might improve the efficiency of therapies against chronic iDCM before heart failure occurs.

## Background

Inflammatory processes usually characterize myocarditis, the progression of which leads to development of dilated cardiomyopathy (DCM) causing heart failure and finally to heart tissue destruction [1,2]. Dilated cardiomyopathy (DCM) is a heart muscle disease which leads to the enlargement of one or both ventricles and consequent systolic dysfunction. DCM is a consequence of persistent exposure to various cellular stress signals, including pro-inflammatory, viral, oxidative, neuro-hormonal, and other micro- or macro environmental factors [3]. Accumulating data showed an importance of inflammatory component in the development of DCM, indicating a relation between myocarditis, autoimmunity and DCM even if immunosuppressive therapy is not always effective [4,5]. Therefore, a better understanding of the molecular mechanisms dominating in inflammatory DCM (iDCM) is needed in order to improve treatment and prevent further heart destruction.

Pro-inflammatory cytokines are not constitutively expressed in the heart but are rapidly expressed in response to cardiac injury [6]. In most cases increased inflammation is in most cases a natural response to injury, helping to heal and recover damaged tissues, whereas overwhelming inflammation aggravates the disease [7]. High levels of accumulated pro-inflammatory factors induce various pathogenic effects, such as improper functioning of the left ventricle, cardiomyocyte death and/or fibrosis [8-10]. It was shown that inflammatory mediators, such as IL-6 and tumor necrosis factor - alpha (TNF-α) can activate apoptotic Fas-Fas ligand pathway in chronic heart failure [11]. Additionally, inflammatory cytokines activate members of the matrix metalloproteinase (MMP) family, zinc-depended endopeptidases, which participate in remodeling of ECM and fibrotic processes [12]. It is agreed, that likewise to heart failure, the main processes characterizing DCM are hypertrophy or loss/death of myocytes and interstitial fibrosis [13]. Despite of that, not all previously mentioned features fit to various forms of DCM. Therefore, in the present study, we have investigated the level of inflammation and subsequent induction of apoptosis, necrosis and fibrosis in sera and biopsies of chronic iDCM patients with purpose to estimate which of previously mentioned processes are mostly and firstly activated. A more detailed estimation of molecular mechanisms will allow more efficient use of therapeutic means to treat chronic iDCM and thereby prevent further myocardium destruction.

## Methods

### Inclusion and exclusion criteria

Study subjects were 32 consecutive patients (25 males, 7 females, mean age 43.14 ± 11.86 years), admitted to a tertiary referral Centre with clinically diagnosed DCM. All patients showed enlarged LV associated with significantly impaired systolic function (LVEF less 45%) on echocardiography in association with long duration of heart failure symptoms. At enrolment, the average duration of observed symptoms was 24–30 month. In addition to clinical severity of heart failure according to the New York Heart Association classification, heart failure was also identified by determination of concentration of brain natriuretic peptide (BNP) in sera.

*Exclusion criteria:* 1) Known causes of heart failure, such as hypertension, significant coronary artery disease, valvular heart diseases, although not relative mitral regurgitation, endocrine diseases, significant renal diseases or drug or alcohol abuse; 2) Acute myocarditis suspected by clinical presentation and diagnostic tests (signs or symptoms of systemic infections; elevated erythrocyte sedimentation rate, reactive C protein level and TnT/TnI, acute chest pain with ST/T wave changes such as: ST-segment elevation or depression and T-wave inversions; new onset of non-specific ECG signs like: bundle branch block, AV-block and/or ventricular arrhythmias; regional wall motion abnormalities on echocardiography; edema and/or LGE on CMR).

**Figure 1:**
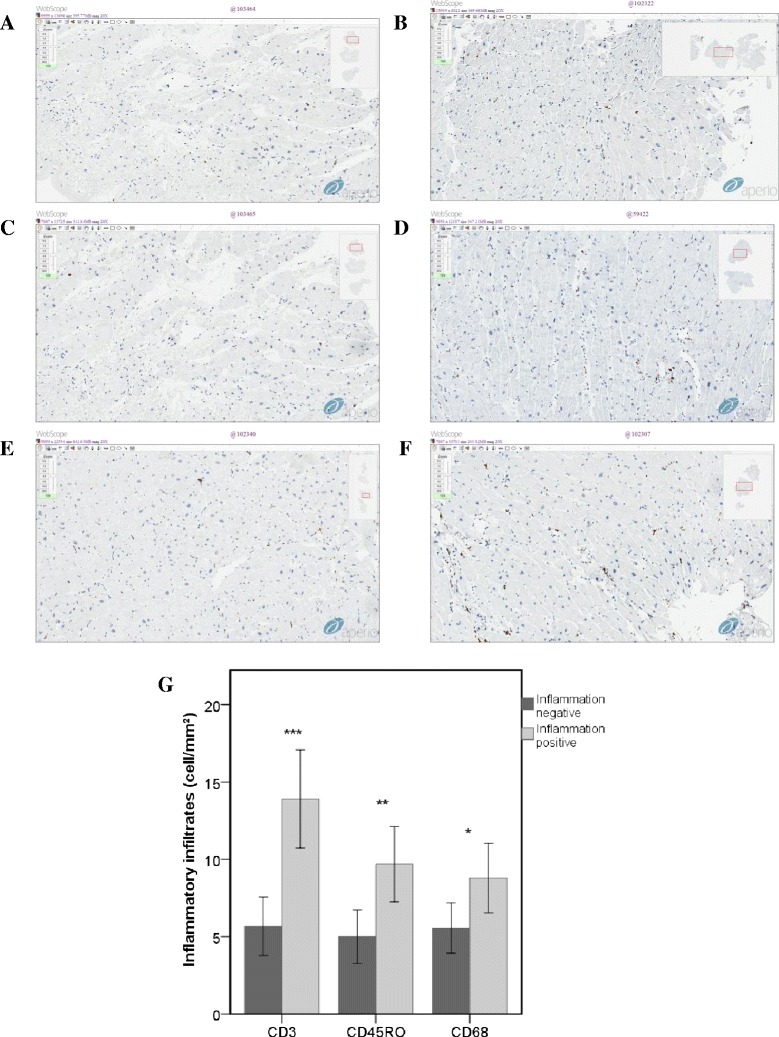
**Immunocytochemistry of inflammatory infiltrates. (A)**. CD3 negative, (3 cell/mm^2^). **(B)**. CD3 positive, (20 cell/mm^2^). **(C)**. CD45Ro negative, (3 cell/mm^2^). **(D)**. CD45Ro positive, CD45Ro (17 cell/mm^2^). **(E)**. CD68 negative, (5 cell/mm^2^). **(F)**. CD68 positive, (20 cell/mm^2^). **(G)**. Mean of total expression of inflammatory infiltrates. Immunohistochemical illustrations are representative and obtained from one inflammatory-positive and one inflammatory-negative patient. ELISA data are presented as means ± SE from al least three independent measurements. Data were considered significant at **p < 0.01 and ***p < 0.001.

All patients signed written informed consent for cardiac catheterization and endomyocardial biopsies (EMB), which includes resulting analysis to elucidate a possible origin of the myocardial and coronary artery diseases. Inflammatory cardiomyopathy was defined as a cardiomyopathy with decreased LVEF, increased LVEDD, and a positive myocardial inflammation score according to the Dallas criteria as well as criteria of the ESC/WHO [14-16]. All patients were subdivided into two groups: inflammation-positive (n = 22) and inflammation-negative (n = 10) according to immunohistochemically proven upregulation of inflammatory infiltrate markers. CD3 (T lymphocyte), CD45Ro (active-memory T lymphocyte) and CD68 (macrophage) were determined immunohistochemicaly by counting positively stained cells per mm^2^. Immunohistochemical stainings (IHC) of collected samples revealed the significant amounts of inflammatory cellular infiltrates (≥14 leucocytes/mm^2^ including up to 4 monocytes/macrophages/mm^2^ with the presence of CD3 positive T-lymphocytes ≥7 cells/mm^2^) in the biopsy samples (Figure 1). A cut off for inflammation samples (diffuse, focal or confluent) and the presence or the absence of necrosis and fibrosis were determined as earlier described [15].

### Additional medical examinations

All patients were interviewed about their medical history and underwent a careful physical examination, as well as laboratory studies, including test of thyroid function, serum electrolytes (sodium, potassium), hsCRP**,** glucose, HbA1c, cholesterol, triglyceride, HDL, LDL, cardiac enzymes (CK, CK-MB, AST), hsTnT, urea, creatinine, uric acid, coagulation tests (PT, aPTT), blood count (hemoglobin, haematocrit, RBC, WBC and platelet count). Each patient underwent investigations such as blood pressure on admission, ECG, echocardiography, Holter monitoring and spiroergometry. The same basic medical treatment scheme was applied to all patients. Essential physical and laboratory data are shown in Table 1.

**Table 1:** **Baseline characteristics of patients**

***Variable***	***Inflammation negative group***		***Inflammation positive group***		
	**Total No. of pts.**	**Value**	**Total No. of pts.**	**Value**	**p Value**
**Sex (male/female)**	10	8 (80%) / 2 (20%)	22	17 (77%)/5 (33%)	0.863¿
**Age (years)**	10	46.7 ± 5.87	22	42.36 ± 2.07	0.389
**NYHA**					
II	10	1 (10%)	22	0 (0%)	0.132¿
III	10	7 (70%)	22	15 (68%)	0.918¿
IV	10	2 (20%)	22	7 (32%)	0.491¿
**Cardiac parameters**					
LBBB (%)	10	3 (30%)	22	5 (22.7%)	0.659¿
Permanent AF (%)	10	2 (20%)	22	0 (0%)	0.000¿*
LVEF (%)	10	24.10 ± 2.28	22	23.05 ± 1.35	0.678
LVEDD (cm)	10	6.89 ± 0.17	22	6.89 ± 0.19	0.998
LVEDDI (cm/m2)	10	3.68 ± 0.21	22	3.71 ± 0.09	0.847
Mean Ao (mmHg)	10	92.00 ± 3.95	22	86.06 ± 2.71	0.291
Mean RAP (mmHg)	10	16.22 ± 3.19	22	11.44 ± 1.74	0.164
Mean PCWP (mmHg)	10	25.00 ± 2.79	22	23.45 ± 2.70	0.731
Mean PAP (mmHg)	10	34.89 ± 4.33	22	32.95 ± 3.24	0.734
CI (L/min/m2)	10	2.38 ± 0.33	22	2.2 ± 0.14	0.573

Data are presented as the means ± SE. *Significant at 0.05 level. ¿Chi- square test. *Abbreviations:* NYHA – New York Heart Association functional class; LBBB – left bundle branch block; AF – atrial fibrillation; LVEF - left ventricular ejection fraction; LVEDD – left ventricular end-diastolic diameter; LVEDDI – left ventricular end-diastolic diameter index; Ao – aortic; RAP – right atrial pressure; PCWP – pulmonary capillary wedge pressure; PAP – pulmonary artery pressure; CI – cardiac index.

### Cardiac catheterization and endomyocardial biopsies

Before EMB, each patient underwent coronary angiography to exclude coronary artery disease as well as right heart catheterization to assess haemodynamic parameters: mean pulmonary artery (PA) pressure, pulmonary capillary wedge pressure (PCWP) and pulmonary vascular resistance (PVR). Right ventricular EMB was obtained using a flexible bioptome via the right femoral vein [17]. Myocardial dilatation was assessed by ultrasound. Biopsies were taken from the right inter-ventricular septum from patients with confirmed absence of ischemia and cardiovascular pathology (stenosis and occlusion). Biopsy specimens were immediately placed at −80°C and later processed for appropriate studies. At least three EMBs samples were subjected to the conventional histological and immunohistochemical evaluation, while two EMBs were stored in a biobank as retained biosamples. Blood collection tubes (8.5 ml) were used for serum sampling of each patient at the same time as EMB.

### Preparation of blood samples

Collected blood samples were placed in vacutainer tubes without anticoagulants and kept at room temperature for 30–45 min to allow clotting. Samples were centrifuged for 15 min at the manufacturer’s recommended speed (1,000 - 2,000 RCF). The upper layer was carefully aspired, checked for turbidity, aliquated into cryovials, labeled and stored at −80°C. Before measurement, all serum samples were thawed on ice, centrifuged at 12,000 g for 5 min and, if necessary, appropriately diluted.

### Biochemical assays of inflammatory infiltrates and cytokines

Inflammatory infiltrates were estimated on fixed, paraffin-embedded material. For classification of biopsies, the previously mentioned markers (Santa Cruz Biotechnology, Inc.), CD3 (T lymphocytes), CD45Ro (active-memory T lymphocytes) and CD68 (macrophages) were applied. Positive cells were registered by an experienced pathologist and expressed as number of cells per mm^2^_._

The pro-inflammatory cytokines TNF-α, IL-6 and IL-1β in serum samples were assayed by solid-phase, chemoluminescent immunometric assays using IMMULITE/Immulite 1000 systems (Immulite, Siemens) according to the manufactures’ instructions: TNF-α (Catalog No: LKNFZ (50 tests), LKNF1 (100 tests); IL-6 (Catalog No: LK6PZ (50 tests), IL-1β (Catalog No: LKL1Z (50 tests)).

### Biochemical assays for apoptosis and necrosis

ELISA was used to assay endomyocardial biopsies and serum samples. The following biomarkers were analyzed: Bcl-2, caspase-9, caspase-8 (Novus Biologicals Europe, Cambridge, UK); Bax (Elabscience Biotechnology Co., Ltd, China); caspase 3, MMP9, TIMP1, APO1/Fas/CD95, FasL (Invitrogen, Paisley, UK) and HSP60 (AssayPro, Saint Charles, Missouri, USA).

All collected serum samples were centrifuged at 12,000 g for 5 min, aliquated in 50 μl portions and stored at −80°C. Before measurement, serum samples were thawed on ice, appropriately diluted and analyzed by ELISA.

Collected heart tissue biopsies were immediately inserted into clean tubes and kept at −80°C. Before measurement, tissue samples were lysed in 100 μl of RIPA lysis buffer (Thermo Scientific Inc., USA), supplemented with protease- and phosphatase mini-inhibitor tablets, 1 mM PMSF, 1 mM Na2VO4 and 25 mM NaF according to the manufacturer’s suggestion (Thermo Scientific Inc., USA). Biopsy samples were sonicated at 10 mV for 2 × 5 s on ice by a Bandelin Sonopuls sonicator, kept for 30 min on ice, centrifuged at 12,000 g for 15 min, aliquoted and stored at −80°C.

Amount of protein in serum and biopsy samples were measured using a modified Lowry Protein Assay kit according to the manufacturer’s recommendations (Thermo scientific Inc., USA). Absorbance was measured with a spectrophotometer (Asys UVM 340 Microplate Reader UK - Biochrom Ltd.) set to 750 nm. The exact protein concentration of each unknown sample was estimated using bovine serum albumin (BSA) as a standard. Total protein concentrations were expressed as μg/ml. Final concentration of biomarkers was expressed as ng/mg of protein or pg/mg protein.

The myocardial injury marker, high-sensitivity troponin T (hsTnT), was measured in serum using an Elecsys 2010 analyzer (Roche Diagnostics, Indianapolis, Indiana) and expressed as pg/ml. Myocardial necrosis was estimated and scored by a competent pathologist on at least three independent routinely stained (Haematoxylin and Eosin (H&E)) sections. Normal myofibres had peripheral nuclei, intact sarcolema and non-fragmented nuclei. Pyknosis of muscle fiber nuclei, edema, and beginning of leuco-diapedesis from the capillaries suggested that the myocardial cells had reached the stage of necrosis.

### Histochemical measurement of fibrosis in endomyocardial biopsies

Tissues collected for histological analyzes were fixed in 10% neutral buffered formalin, and then paraffin-embedded in a tissue processor. Total cardiac fibrosis (including interstitial and perivascular forms) was assessed. Specimens were stained with Masson’s trichrome connective tissue stain according to a standard protocol. Keratin and normal muscle fibers stained red, whereas fibrotic areas stained blue. Digital images from the experimental glass slides were obtained using ScanScope Digital Slide Scanner (Aperio, Vista, CA) at a 20× magnification and archived on a devoted Spectrum Server 11.1.0.751 (Aperio). Quality control of the scanned images and all further analysis were performed using ImageScope V11.1.2.760 (Aperio) and WebScope V11.1.0.756 (Aperio).

Digital analysis of the slides was done using a Colocalization V9 algorithm that was run for the whole slides, ignoring the number of tissue cross sections on it – making the process fully automated. A colocalization algorithm uses the deconvolution method to separate the stains and classifies each pixel according to the number of stains present. For colocalization, the threshold for each stained sample is specified for a required stain (e.g. Masson’s trichrome) and the algorithm reports the percentage of areas for each stain combination is detected: 1, 2, 3, 1 + 2, 1 + 3, 2 + 3, 1 + 2 + 3 or none (up to 3 areas were analyzed). The algorithm also provides an eight-color mark-up image for visualization of colocalized stains. Summing up the stain combinations 3, 2 + 3 and 1 + 3 calculated the total percentage of cardiac fibrosis.

### PCR assay

Intramyocardial viruses were estimated by PCR assay [18]. Briefly, genomic DNA and total RNA were extracted simultaneously using ZR-Duet™ DNA/RNA Miniprep kit (Zymo Research, Irvine, CA, USA). RNA (1 μg) was reversely transcribed in 20 μl reaction volumes using random hexamers and First Strand cDNA Synthesis Kit (Thermo Fisher, Vilnius, Lithuania) according to the vendor’s recommendations. Forward primers for the second round of PCR were labeled with 6-carboxyfluorescein (FAM) at the 5′ end. Final PCR products were 10-fold diluted and analyzed by capillary electrophoresis on a Genetic Analyzer 3130*xl,* using GeneScan™ 600 LIZ™ Size Standard and Gene Mapper Software v4.1 (Applied Biosystems, Foster City, CA, USA) for sizing PCR fragments. The following virus species were detected in the biopsies of 16 patients: parvo virus B19 (n = 11), human herpes virus type 6 (n = 4), hepatitis C virus (n = 1), Eppstein-Barr virus (n = 1), entero virus (n = 1), and varicella zoster virus (n = 1). The frequencies of detected viral genomes were equal in each tested group (50%) and, therefore, it did not influence the results.

### Statistical analysis

All statistical analyses were performed using the SPSS package (version 19.0 for Windows; SPSS Inc., Chicago, IL, USA) at not higher than 5% significance level. The normality of the data distribution was tested by the Shapiro-Wilk test. Significance of measurements was tested by Student’s *t* test or the Wilcoxon–Mann–Whitney rank sum non-parametric test. For comparative purposes Pearson’s correlation coefficient was used.

### Ethical approval

The study was approved by the local Lithuanian Bioethics Committee (license No. 158200-09-382-l03; No. 158200-382-PP1-23). All patients signed written informed consent to include their data in the study for each investigational procedure. The investigation conforms to the principles outlined in the *Declaration of Helsinki*.

## Results

### Inflammatory markers in dilated cardiomyopathy

Here, we monitored the inflammatory process by detecting expression of CD3, CD45Ro and CD68 in inflammatory infiltrates by immunohistochemistry (Figure 1). In addition, we determined levels of inflammatory cytokines TNF-α, IL-6 and IL-1β in sera. Representative immunohistochemical micrographs show the increased and diffused expression of CD3, CD45Ro and CD68 (Figure 1A, B, C, D, E and F). Total expression of cytokines in infiltrates from inflammatory-negative and -positive groups is shown in Figure 1G. T-lymphocyte receptors (CD3) and active memory T-lymphocytes (CD45Ro) were significantly upregulated 2.38-fold (*P* < 0.001) and 2.1-fold (*P* < 0.01), respectively (Figure 1G). Significant accession of CD3 and CD45Ro in iDCM myocardium also suggests increased myocardial micro-vascular permeability.

Our data showed upregulation of the specific and general inflammatory markers interleukin-6 (IL-6) and C-reactive protein (hsCRP), respectively, in iDCM serum samples (3.45-fold, *P* < 0.05 and 2.76-fold) (Table 2). Changes of tumor necrosis factor alpha (TNF-α) and interleukin-1beta (IL-1β), also known as catabolin, were not significant in the iDCM sera.

**Table 2:** **Summarized data of measured biomarkers**

***Variable***	***Inflammation negative group***		***Inflammation positive group***		
	**Total No. of pts.**	**Value**	**Total No. of pts.**	**Value**	**p Value**
**Markers of inflammation in serum**					
TNF-α (pg/mL)	8	7.9313 ± 0.5106	21	14.2819 ± 5.0280	0.223
IL-6 (pg/mL)	8	3.3938 ± 0.8554	21	11.4038 ± 3.3614	0.031*
IL-1β (pg/mL)	8	5.0000 ± 0.0000	21	4.7619 ± 0.2381	0.329
CRP (μg/mL)	8	7.6875 ± 5.0460	19	21.5563 ± 6.9633	0.066**¡**
**Markers of apoptosis in serum**					
Bcl2 (ng/mg protein)	10	0.0288 ± 0.0288	22	0.0536 ± 0.0455	0.889**¡**
Bax (ng/mg protein)	10	2.152717 ± 0.24	22	2.3354 ± 0.1606	0.535
Caspase-9 (ng/mg protein)	10	0.012955 ± 0.0013	22	0.0808 ± 0.0283	0.038*
Caspase-8 (ng/mg protein)	10	0.001 ± 0.0001	22	0.0031 ± 0.0009	0.043*¡
Caspase-3 (ng/mg protein)	10	0.0029 ± 0.0022	22	0.0105 ± 0.0023	0.025*
APO1/Fas/CD95 (ng/mg protein)	10	0.0000 ± 0.0000	22	0.00004 ± 0.00004	0.857**¡**
FasL (ng/mg protein)	10	0.0000 ± 0.0000	22	0.0000 ± 0.0000	N.A.
HSP60 (ng/mg protein)	10	0.0419 ± 0.0253	22	0.3760 ± 0.1468	0.035*
**Markers of apoptosis in biopsy**					
Bcl2 (ng/mg protein)	10	83.5523 ± 26.2936	21	63.8790 ± 17.2137	0.540
Bax (ng/mg protein)	10	5.6452 ± 2.6905	21	6.8873 ± 3.7924	0.724**¡**
Caspase-9 (ng/mg protein)	10	29.6575 ± 12.5969	21	38.7122 ± 9.6108	0.950**¡**
Caspase-8 (ng/mg protein)	10	0.9483 ± 0.1640	21	1.1611 ± 0.1962	0.413
Caspase-3 (ng/mg protein)	10	0.2503 ± 0.0773	21	0.2586 ± 0.0649	0.935
APO1/Fas/CD95 (ng/mg protein)	10	3.4651 ± 0.6568	21	4.1921 ± 0.6607	0.443
FasL (ng/mg protein)	10	4.5550 ± 1.3594	21	4.0588 ± 1.1083	0.780
HSP-60 (ng/mg protein)	10	24.1262 ± 6.9102	21	19.2656 ± 4.5617	0.565
**Marker of heart tissue contraction in serum**					
hsTnT (pg/mL)	8	35.4988 ± 9.0908	20	66.4145 ± 26.9755	0.289
**Markers of extracellular matrix degradation in serum**					
MMP9 (ng/mg protein)	10	1.3867 ± 0.0674	22	1.5261 ± 0.0508	0.115
TIMP1 (ng/mg protein)	10	5.9610 ± 0.3597	22	6.1223 ± 0.1497	0.686
MMP9/TIMP1	10	0.2355 ± 0.0090	22	0.2511 ± 0.0086	0.223
**Markers of extracellular matrix degradation in biopsy**					
MMP9 (ng/mg protein)	10	2.3698 ± 1.1931	21	2.7630 ± 0.9394	0.798
TIMP1 (ng/mg protein)	10	9.4917 ± 1.7605	21	7.8056 ± 1.4029	0.462
MMP9/TIMP1	10	0.1931 ± 0.0729	21	0.4760 ± 0.1048	0.035*
Frequency of viral genome	5	50%	11	50%	
BNP (pg/mL)	10	1277.8500 ± 428.5054	22	1603.2591 ± 276.3777	0.532

Data are presented as the means ± SE. *Significant at 0.05 level. **¡** Wilcoxon–Mann–Whitney rank sum nonparametric test.* Abbreviations:* TNF-α - tumor necrosis factor α; IL-6 – interleukin-6; IL-1β – interleukin 1β; Bcl-2 – B-cell lymphoma 2 protein; Bax – Bcl-2–associated X protein; Hsp60 – heat shock protein 60; MMP9 – matrix metalloproteinase 9; TIMP1 – tissue inhibitor of matrix metalloproteinase 1; TNF-a – tumor necrosis factor-alfa; IL-1β – interleukin 1 beta; IL-6 – interleukin 6; hs TnT – high sensitivity troponin T; CRP – C-reactive protein, BNP- B-type natriuretic protein; N.A. – not available.

### Changes of apoptotic and necrotic biomarkers in iDCM samples

**Figure 2:**
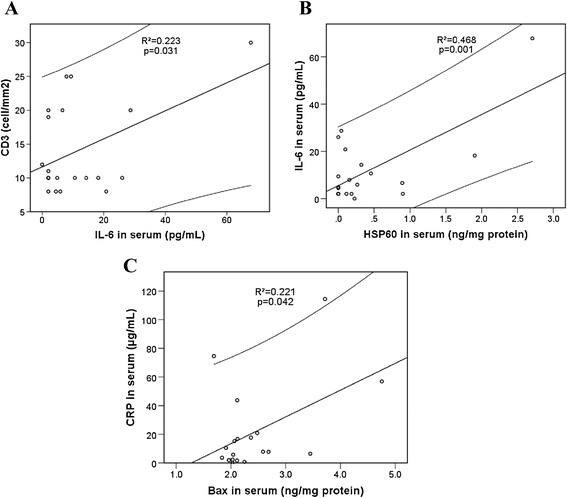
**Correlation between inflammatory and mitochondrial membrane destabilization markers. (A)**. Correlation between serum inflammatory cytokine IL-6 and CD3. **(B)**. Correlation between IL-6 and mitochondrial membrane stabilizing chaperone Hsp60 in serums. **(C)**. Correlation between C-reactive protein (CRP) and Bax in serums. Correlation analysis was done by the statistical SPPS programme. Correlation was significant at a level of *P* <0.05. Linear regression line is presented within 95% confidence interval. Regression coefficients (R^2^) are shown in the graphs.

Data presented in Figure 2A show significant correlation between CD3 and IL-6. Significant correlation of IL-6 and hsCRP with the mitochondrial chaperonic protein Hsp60 and pro-apoptotic Bax in sera, respectively, suggests that myocardial inflammation mostly affected the integrity of mitochondrial membranes (Figure 2B and C). Levels of the mitochondrial membrane stabilizing protein Hsp60 in inflammation-positive sera were 8.97-fold higher (*P* < 0.05). Changes of APO1/Fas/CD95 (FasR), the main receptor of extrinsic apoptotic pathway, and its ligand (FasL) in sera and biopsies were not significant (Table 2).

**Figure 3:**
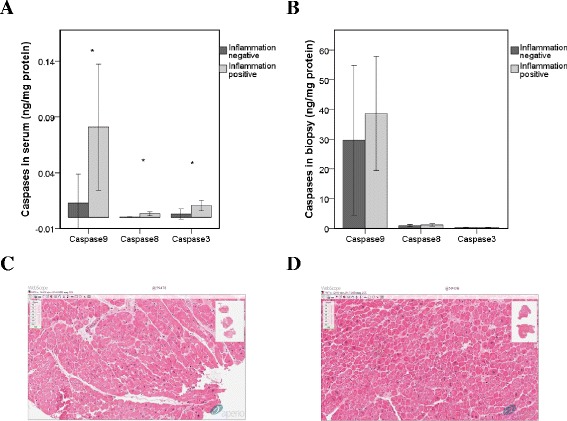
**Levels of pro-caspases-9, −8, and −3 in iDCM samples. (A)**. Levels of caspases in iDCM serum samples. **(B)**. Levels of caspases in iDCM biopsies. **(C)**. Histological estimation of necrosis in inflammation-negative EMB. **(D)**. Histological estimation of necrosis in inflammation-positive EMB. Images are representative from one EMB of each group. Data are presented as means ± SE from at least three independent measurements. Data were considered significant at *P < 0.05.

Data presented in Figure 3A demonstrate statistically significant (*P* < 0.05) increase of caspase-9, −8 and −3 in sera with the most prominent expression of caspase-9. Enhanced expression of the same caspases in endomyocardial biopsy samples (Figure 3B) was, however, slight and insignificant. The upregulation of high sensitivity troponin T (hsTnT), a major structural sarcomeric protein of the heart and a marker of necrosis and/or cardiomyocyte injury, in sera was not significant (Table 2). However, the sarcomeric protein hsTnT in iDCM sera strongly correlated with the levels of caspases-8, Bax and caspase-3 in biopsies, suggesting cardiomyocyte injury and caspase-regulated release of hsTnT (Table 3).

**Table 3:** **Correlation of apoptotic, necrotic and inflammatory biomarkers in EMB**

	**Casp-9 in biopsy**	**Casp-8 in biopsy**	**Casp-3 in biopsy**	**Bcl2 in biopsy**	**FasR in biopsy**	**FasL in biopsy**	**MMP9 in biopsy**	**TIMP1 in biopsy**	**Bax in biopsy**	**IL-6 in serum**
Casp-8 in biopsy	0.303									
Casp-3 in biopsy	0.063	0.436*								
Bcl2 in biopsy	−0.202	0.175	0.486*							
FasR in biopsy	−0.097	−0.074	0.526*	0.739**						
FasL in biopsy	−0.046	0.007	0.442*	0.835**	0.907**					
MMP9 in biopsy	−0.229	0.024	0.419	0.764**	0.730**	0.824**				
TIMP1 in biopsy	−0.012	−0.205	0.213	0.517*	0.795**	0.722**	0.401			
Bax in biopsy	0.283	0.584**	0.678**	0.056	0.139	0.053	0.128	−0.127		
IL-6 in serum	0.518*	−0.016	−0.011	−0.262	−0.202	−0.154	−0.041	−0.227	0.131	
hs TNT in serum	0.434	0.598**	0.563*	−0.125	−0.067	−0.165	−0.120	−0.249	0.954**	0.231

Two tailed significance: *P < 0.05; **P < 0.01. Significant correlations are in bold type.

*Abbreviations:* Casp-3 – Caspase-3; Casp-8 – Caspase-8; Casp-9 – Caspase-9; IL-6 – Interleukin-6; Bcl-2 – B-cell lymphoma 2 protein; FasR—Fas receptor; FasL – Fas ligand; MMP9 – matrix metalloproteinase 9; TIMP1 – tissue inhibitor of matrix metalloproteinase 1; Bax – Bcl-2–associated X protein; Hsp60 – heat shock protein 60; hsTnT – high sensitivity Troponin T.

### The correlation analysis of apoptotic biomarkers in iDCM sera and biopsies

**Figure 4:**
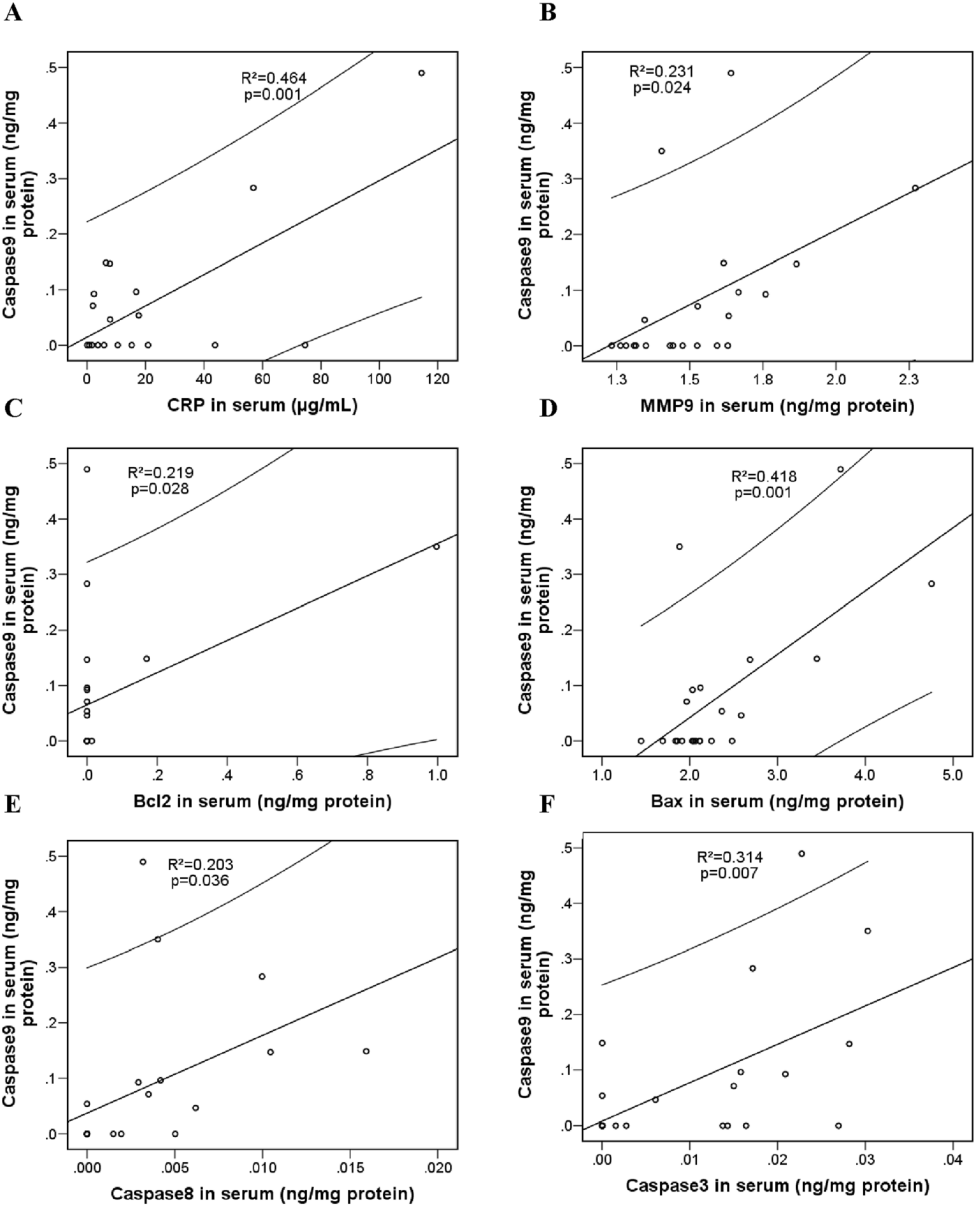
**Correlation of caspase-9 with biomolecules in serum samples.** Caspase-9 correlated with: **(A)**. C-reactive protein (CRP). **(B)**. matrix metalloproteinase-9 (MMP-9). **(C)**. B-cell lymphoma 2 protein (Bcl-2). **(D)**. Bcl-2–associated X protein (Bax). **(E)**. Caspase-8. **(F)**. Caspase-3. Correlation analysis was done by the statistical SPPS programme. Linear regression line is presented within 95% confidence interval. Regression coefficients (R^2^) and statistical significance (*P* < 0.05) are shown in the graphs.

**Figure 5:**
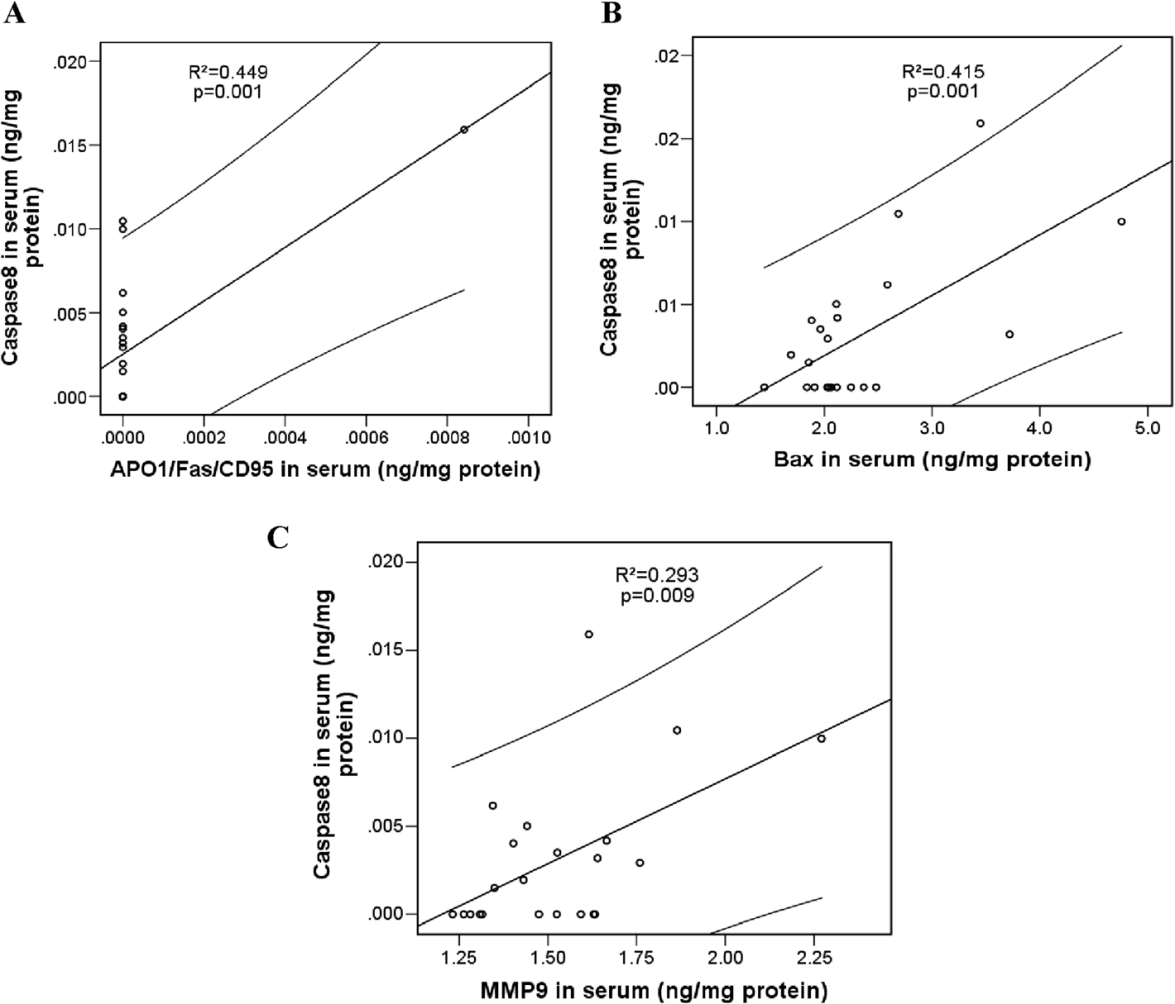
**Correlation between caspase-8 and biomolecules in serum samples.** Correlation of caspase-8 with: **(A)**. Fas receptor (APO1/Fas/CD95). **(B)**. Bcl-2–associated X protein (Bax). **(C)**. Matrix metalloproteinase-9 (MMP-9). Correlation analysis was done using SPPS program. Linear regression line is presented within 95% confidence interval. Coefficients of regression (R^2^) and statistical significance (*P* < 0.05) are shown in the graphs.

Data in Figure 4A demonstrate that caspase-9, a serum cysteine-aspartic acid specific protease, also named apoptosis-initiating caspase, significantly correlated with the general inflammatory marker C-reactive protein (hsCRP) confirming the sensitivity of the intrinsic apoptotic pathway to inflammation. In parallel, the significant correlation between caspase-9 and MMP9 tells us that caspase-9 might be either directly activated by the MMP9 or, alternatively, through the other mediators of the intrinsic apoptotic pathways, such as Bcl-2 and Bax (Figure 4C and D; *P* < 0.05). Results presented in Figure 4E and F show that levels of caspase-9 are strongly correlated (*P* < 0.05) with the executing caspase-3 and extrinsic apoptotic pathway-initiating caspase-8, suggesting the interaction between the intrinsic and extrinsic apoptotic pathways. Furthermore, a significant correlation was observed between caspase-8 and the APO1/Fas/CD95 levels in iDCM sera (Figure 5A). Caspase-8 also significantly correlated with the pro-apoptotic Bax and MMP9 confirming the intersection of extrinsic and intrinsic pathways at mitochondrial level and caspase-8 activation by MMP9 (Figure 5B and C).

Next, we have investigated correlation of apoptotic biomarkers in iDCM myocardium. Caspase-9 in heart tissues, similarly to that in sera, significantly correlated with pro-inflammatory cytokine IL-6, and caspase-8 in myocardium correlated with Bax and caspase-3 (Table 3). The executing caspase-3 also demonstrated significant correlation with both the intrinsic (Bax, Bcl-2) and extrinsic (APO1/Fas/CD95 and FasL) apoptotic pathways (Table 3). Similarly to that in sera, members of both apoptotic pathways (Bcl-2, APO1/Fas/CD95 and FasL) in biopsies significantly correlated with MMP9 and its inhibitor TIMP1 (Table 3).

### Estimation of fibrosis in iDCM biopsies

**Figure 6:**
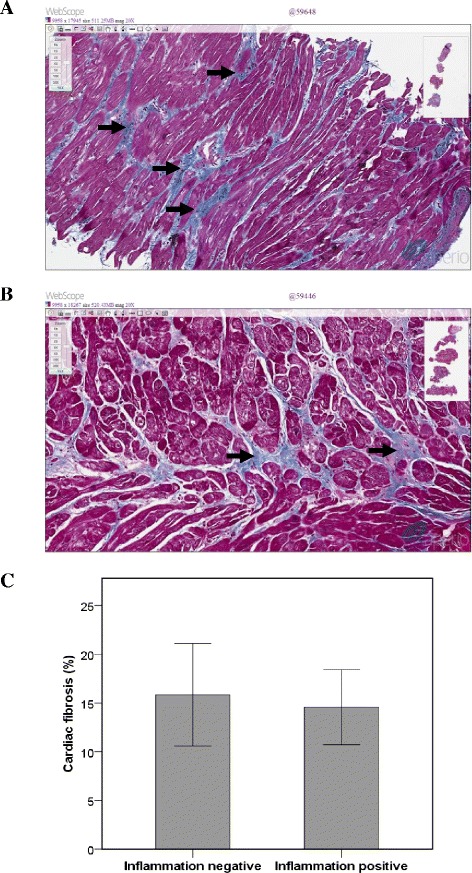
**Histopathological findings of fibrosis in right ventricular EMB. (A)**. Inflammation-negative EMB. **(B)**. Inflammation-positive EMB. **(C)**. Quantitative expression of fibrosis. Micrographs show one representative picture from one patient of each group. Fibrosis is colored blue. Magnification × 10.

The final experimental part was dedicated to investigate the level of fibrosis in the EMB samples. Data in Figure 6A and B show that the level of fibrosis (blue stain) in inflammatory-positive biopsies was slightly lower compared to the inflammatory-negative ones. The quantitative expression of fibrosis staining confirmed our observation (Figure 6C). The exact roles of collagen I and III in fibrosis were impossible to evaluate due to the absence of collagen I and III quantification algorithm under polarized light. This part needs additional investigations.

## Discussion

Inflammatory cardiomyopathy is often defined by myocarditis in association with cardiac dysfunction and inflammation as a main factor of interconnection [15,19]. Long-term studies have shown that less than 15% of patients with acute myocarditis developed dilated cardiomyopathy [20]. It was also demonstrated that patients with proved active myocarditis in biopsies and idiopathic DCM had the same long-term outcome (56% vs. 54%, respectively) [21]. Therefore, investigation of underlying basic molecular mechanisms and their possible pharmacological modulation could improve treatment of chronic iDCM.

Formerly the inflammatory markers were thought to be only indicators of risk but not causal factors [22]. However, it was shown that the pro-inflammatory cytokines, such as TNF-α, IL-6 and IL-1β, might act synergistically at both mRNR and protein levels by impairing cardiac contraction [23,24]. Pro-inflammatory IL-6 has been reported to be involved in the remodeling of left ventricle after myocardial infarction and induced heart failure leading to skeletal muscle atrophy and heart disorders [25,26]. The molecular mechanisms by which IL-6 affect myocardium can be related to a negative ionotropic effect and upregulation of nitric oxide synthase, downregulation of SERCA2, stimulation of collagen synthesis to name a few [27-29]. The activation of molecular mechanism in heart usually depends on the origin, duration and intensity of toxic stimuli.

Recently, a strong and direct influence of IL-6 was shown on mitochondrial function: IL-6 inhibited adipocyte mitochondrial membrane potential, ATP production and increased intracellular ROS levels [30,31]. Similar to IL-6, a general inflammatory biomarker C-reactive protein (hsCRP) also correlated with poor DCM prognosis, heart failure and mitochondrion-mediated myocyte apoptosis [32-34]. It was also shown that, CRP contributes to IL-6 expression which is a stronger prognostic predictor of heart failure than CRP [35,36]. In agreement with previous observations, our data show significant upregulation of IL-6 in chronic iDCM sera and its significant correlation with numbers of infiltrated T-lymphocytes (CD3) and secreted intra-mitochondrial Hsp60 protein levels. The significant correlation of CRP with pro-apoptotic Bax and strong upregulation of caspase-9 in sera confirmed that inner apoptotic pathway is more sensitive to inflammation compared to the extrinsic one. Additionally, the absence of necrosis and insignificant upregulation of apoptosis markers in biopsies suggest the beginning of cell death in chronic iDCM myocardium.

Many recent findings demonstrate interaction between both apoptotic pathways strengthening toxic effects in heart [37]. It was shown that the member of the extrinsic apoptotic pathway pro-caspase-8 cleaves the BH3 domain-only protein Bid, which in turn, activates Bax, stimulates its integration into mitochondrial membranes and release of cytochrome *c* [38,39]. In agreement with previous observations, data of the present study show a significant correlation between caspase-8 and Bax levels in iDCM sera, suggesting that Bax can be one of the most important intersection points between the intrinsic and extrinsic apoptotic pathways. However, the mediators of extrinsic apoptotic pathway in iDCM samples had low initial level and intensity of activation suggesting this pathway being a supporter but not a main leader of heart cell death. The other authors’ observations confirmed that the extrinsic apoptotic pathway might have low impact on cardiomyocyte death in left ventricular tissue [40].

The ECM is an important mediator between different cell types within the myocardium supporting its structural network which can be degraded by proteolytic enzymes matrix metalloproteinases (MMPs). In animal model, a time-dependent increase of myocardial MMPs levels were related to the progression of left ventricular dilation and dysfunction [41]. Similar to the intrinsic apoptotic pathway, MMP-9 was also found to be sensitive to inflammation and to participate in the pathogenesis of cardiomyopathy [42]. Additionally, it was shown that chronic heart failure involves endothelial apoptosis in response to MMP-9 activation [43]. Data of this study showed that the MMP-9/TIMP1 ratio in iDCM biopsies was significantly upregulated and significantly correlated with the markers of both apoptotic pathways (Bcl-2, Fas receptor and Fas ligand). These observations suggest that upregulation of MMP-9/TIMP1 are more related to the activation of apoptotic pathways than to the stimulation of fibrosis. Parallelly, the significant correlation between hsTnT and apoptotic proteins caspases-8, Bax and caspase-3 also reveals hsTnT contribution to the impairment of cardiomyocyte and/or contraction ability by activation of pro-apoptotic signaling cascades.

## Conclusions

The present study demonstrates that persistent myocardial stressors increase T-lymphocytes in myocardium of iDCM patients which correlates with the augmented secretion of inflammatory cytokines, such as IL-6. Chronic myocardial inflammation subsequently affects mitochondria and induces significant release of Hsp60 and caspase-9, −8 and −3, suggesting activation of both apoptotic pathways with stronger implication on the intrinsic pathway. The pro-apoptotic Bax is an important intersection point for the extrinsic- and intrinsic apoptotic pathways, blockage of which might improve iDCM treatment. The significant activation of MMP-9/TIMP1 was enough to support apoptosis but not fibrosis.

Taken together, our study suggests that biomarkers secreted to the serum parallels intramyocardial processes and, therefore, might be useful not only for the diagnosis but also for detailed studies of the molecular mechanism behind chronic iDCM. The observed absence of necrosis and fibrosis in chronic iDCM shows that disease had still not reached an end-stage and might be controlled by anti-inflammatory and specific anti-apoptotic drugs.

### Limitations

The main limitation of this study is that it contains a relatively low number of iDCM patients. The study also lacks normal heart biopsies because of ethical reasons. However, the main goal of this study was to investigate the main processes, molecular mechanism and signaling pathways dominating in chronic iDCM of unknown origin. The collection of additional iDCM samples continues and, hopefully, in the future we will be able to understand more about iDCM diagnosis and its treatment.
